# Air Pollution and Myocardial Infarction—A New Smoker’s Paradox?

**DOI:** 10.3390/jcm13237324

**Published:** 2024-12-02

**Authors:** Friederike von Lewinski, Franz Quehenberger, Michael Sacherer, Valentin Taucher, Christoph Strohhofer, Klemens Ablasser, Nicolas Verheyen, Caren Sourij, Andreas Kainz, Gerit Wünsch, Andrea Berghold, Thomas M. Berghaus, Sadeek Sidney Kanoun Schnur, Andreas Zirlik, Dirk von Lewinski

**Affiliations:** 1 Division of Endocrinology, Department of Internal Medicine, Medical University of Graz, 8036 Graz, Austria; friederike.von-lewinski@medunigraz.at; 2 Institute for Medical Informatics, Statistics and Documentation, Medical University of Graz, 8036 Graz, Austria; franz.quehenberger@medunigraz.at (F.Q.); andreas.kainz@medunigraz.at (A.K.); gerit.wuensch@medunigraz.at (G.W.); andrea.berghold@medunigraz.at (A.B.); 3 Division of Cardiology, Department of Internal Medicine, Medical University of Graz, 8036 Graz, Austria; michael.sacherer@medunigraz.at (M.S.); valentin.taucher@medunigraz.at (V.T.); christoph.strohhofer@stud.medunigraz.at (C.S.); klemens.ablasser@medunigraz.at (K.A.); nicolas.verheyen@medunigraz.at (N.V.); caren.sourij@medunigraz.at (C.S.); sadeek.kanoun-schnur@medunigraz.at (S.S.K.S.); andreas.zirlik@medunigraz.at (A.Z.); 4 Department of Cardiology, Respiratory Medicine and Intensive Care, University Hospital Augsburg, University of Augsburg, 86156 Augsburg, Germany; thomas.berghaus@uk-augsburg.de; 5 Doctoral School of Clinical Medicine, Faculty of Medicine, University of Szeged, 6720 Szeged, Hungary; 6 Department of Cardiology, Royal Devon University, Healthcare NHS Foundation Trust, Exeter EX2 5DW, UK

**Keywords:** myocardial infarction, ambient air pollution, smoking, heart failure, diabetes mellitus

## Abstract

**Background/Objectives:** Ambient air pollution is a significant public health concern, known to affect cardiovascular health adversely. Research has identified both long-term and short-term cardiovascular risks associated with various air pollutants, including those linked to acute coronary syndromes. However, the observed effects are rather small, with most data sourced from highly polluted regions. **Methods:** This study utilized a prospective registry database, documenting 12,581 myocardial infarction (MI) events in Styria, Austria from January 2007 to December 2015. Pollutants analyzed included particulate matter (PM_2.5_, PM_10_) and gases, such as NO_2_, CO, SO_2_, O_3_ and NOx. We employed generalized linear models to examine the interaction of each of these pollutants on the daily incidence of MI. Additionally, we conducted separate analyses for patients with specific comorbidities: diabetes mellitus (DM), arterial hypertension (HTN), heart failure with reduced ejection fraction (HFrEF), chronic obstructive pulmonary disease (COPD) and current smokers. **Results:** No significant associations were identified between any of the pollutants and MI incidence, both in the overall cohort and in patient subgroups with DM, HTN, HFrEF or COPD. However, among active smokers, we observed a decreased relative risk of MI associated with elevated levels of NO_2_, CO, SO_2_ and NOx on the day of MI (*p* < 0.01 for all pollutants). Conversely, an increased MI risk was associated with rising ozone levels (*p* = 0.0027). This counterintuitive finding aligns with previously published data and may suggest a new dimension to the “smoker’s paradox”. **Conclusions:** In regions with low pollution levels, air pollutants pose only minor or insignificant short-term risks for myocardial infarction. Active smokers exhibit an altered response to ambient air pollution.

## 1. Introduction

Air pollution has been associated with cardiopulmonary disease, both of which are significant public health concerns. Among the various air pollutants, small particulate matter (PM) has been of particular interest. PM is defined as airborne liquid droplets or solid particles primarily derived from human activities and, to a lesser extent, natural sources. PM can be categorized into PM_10_ and PM_2.5_. PM_10_ consists of particles less than 10 μm in diameter, primarily originating from wildfires, construction-related pollution and mining activities [1]. PM_2.5_ includes finer particles less than 2.5 μm in diameter, which are notably more hazardous and have been identified as the principal contributors to PM-related effects on human and animal health. PM_2.5_ stems mostly from industrial activities and transportation-related fuel combustion. Within this category, ultrafine particulate matter (UFPM), which are particles with a diameter of less than 0.1 μm, is substantially produced by diesel combustion [2]. Unlike PM_10_, which are too large to cross the alveolocapillary membrane, PM_2.5_ and smaller particles can translocate into the bloodstream, exerting systemic organ damage [1]. Recently, the World Health Organization revised its air quality guidelines based on newer evidence and recommended tightening the annual PM_2.5_ threshold to 5 μg/m^3^ from the previous limit of 10 μg/m^3^.

Long-term exposure to air pollution has been linked to an increase in myocardial infarction incidences [3] as well as non-accidental death due to metabolic and cardiovascular complications [4]. Elevated exposure to PM has been shown to adversely impact post-MI mortality during both short-term and mid-term follow-up periods [5], possibly via pro-inflammatory pathways [6].

In a Swedish study, patients with cardiovascular risk factors exhibited an increased risk associated with elevated PM levels. This association was even more pronounced with coronary calcium scores, a marker of subclinical coronary atherosclerosis, highlighting the importance of cardiovascular risk factors in this context [7]. Furthermore, among patients with non-obstructive coronary artery disease, increased particulate levels were positively associated with myocardial infarction with non-obstructive coronary arteries (MINOCA), in contrast to patients without elevated markers of myocardial ischemia.

Additionally, a substantial body of data supports the association between short-term exposure to elevated concentrations of PM and increased rates of myocardial infarction.

MI-associated mortality correlated with PM_2.5_ and PM_10_ concentrations on the day of death and the preceding day, according to a large study from Hubei, China [8]. Similarly, a short-term association with PM_2.5_ was observed in a large Japanese cohort. However, this association was detectable only in the spring, not in other seasons, and was limited to exposures within four days prior to the MI. Notably, the association was no longer statistically significant when a lag-time of five days was analyzed. Interestingly, this association was more pronounced in MINOCA patients compared to those with concurrent coronary artery disease [9]. A recent meta-analysis also reported that short-term exposure to particulate matter significantly increases the risk of various cardio-respiratory diseases, with myocardial infarction being the most notably affected [10].

With respect to other air pollutants, an early meta-analysis attributed a 1 to 5% increased risk of myocardial infarction to short-term exposure to CO, NO_2_ and SO_2_ [11]. However, ozone levels were not significantly associated with increased risk. More recent studies have indicated that NO_2_ has the largest impact, more so than CO, SO_2_ or O_3_ [8,10].

The association between PM and STEMI tends to be more pronounced in patients with comorbidities such as hypertension, diabetes and hyperlipidemia and in those aged over 65, particularly females [12]. However, a separate study in China observed no association between air pollutants and age in over 25,000 MI patients aged over 65 [13]. Moreover, a study in Taiwan demonstrated that the risk increases significantly when three or more risk factors are present, suggesting that the cumulative effect of multiple comorbidities can substantially elevate the risk [14].

Smoking status as a risk factor has only been investigated in a small subset of studies, yet it garners particular interest due to the widely discussed “smoker’s paradox”, which historically noted smaller infarct sizes in smokers. Recent investigations have suggested that differences in baseline characteristics between smokers and non-smokers are the most likely explanation for these discrepancies, leading to a decline in the use of the term “smoker’s paradox” in contemporary research [15,16,17,18]. Interestingly, the negative impact of ambient air pollution appears to be more pronounced in non-smokers [12,19]. However, this observation was not observed during Asian dust episodes, according to a recent Korean study [20].

## 2. Methods

We utilized a prospective registry database containing records from all patients referred to the three catheterization laboratories (cathlabs) in Styria, Austria for acute myocardial infarction between January 2007 and December 2015. Data were first accessed on 6 June 2021 for research purposes. The analysis included 12,581 events from 11,591 patients, after excluding repetitive coronary angiographies (*n* = 134) and coronary angiographies delayed by more than seven days post-MI (*n* = 850). Baseline characteristics of the entire cohort, as well as specified subgroups, are detailed in Table 1. The state of Styria spans 16,401 km² and has a population of approximately 1.25 million.

Patients were classified based on electrocardiographic and biomarker criteria into ST-segment elevation MI (STEMI) and non-ST-segment elevation acute coronary syndromes (NSTE-ACS).

Ambient air pollution values, including levels of PM_2.5_, PM_10_, NO_2_, CO, SO_2_, O_3_ and NOx, were provided by the the Referat Luftreinhaltung in Graz, Austria.

### Statistical Analysis

The yearly and weekly periodicity of MI events was assessed using boxplots. We established a Poisson regression model for each air pollutant and concomitant disease to analyze the daily counts of MI in patients with and without the risk factor. The air pollutants, with the exception of ozone, underwent logarithmic transformation in order to temper the influence of extreme values. Next, they were standardized to a mean of zero and a standard deviation one in order to scale them equally. The regression parameters included a smooth function of date, the day of the week, a sine and cosine function with a one year period, the logarithm of the pollutant (except ozone, as its distribution was not right-skewed and contained zero values), the risk factor indicator and an interaction variable. This interaction variable was defined as the median for patients without the risk factor and the pollutant for patients with the risk factor, assessing how the pollutant’s effect varies between the two groups. A smooth function of the logarithm of the pollutant and the interaction was also calculated to properly assess nonlinear effects. The predicted values and the confidence limits of the interaction effect were displayed in diagrams. 

To align with similar research on the disease risk from air pollution, we calculated a conditional logistic case-crossover model of disease risk. For each MI, the pollution levels on the same days of the week of the calendar month were contrasted with those on the day of the MI event. The intention was to compare the event’s timing to almost identical time points without an event. This is equivalent to a Poisson model in which the smooth function of date, day of the week and sine and cosine functions are replaced by stratification on the month of the year and day of the week [21]. 

The analysis was conducted using R 4.3.1 (www.r-project.org) and the packages mgcv 1.9-0, gnm 1.1-5 and survival 3.5-8, with the smoothing parameter determined by cross-validation. *p*-values below 0.05 were considered to be significant. As there were 42 combinations of risk factors and air pollutants, *p*-values underwent a multiplicity correction using the FDR method, according to Benjamini and Hochberg.

This study was approved by the ethics committee of the Medical University of Graz (project number: 28-433 ex 15/16). Consent was waived by the EC for collecting and analyzing the data. 

## 3. Results

**Figure 1:**
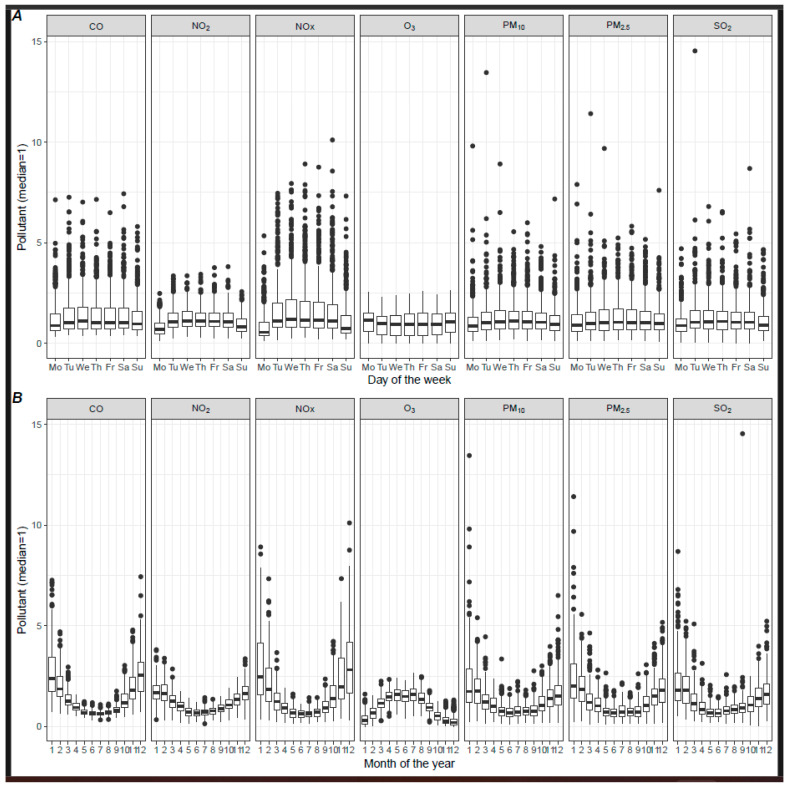
(**A**) Pollutant and day of the week. (**B**) Daily pollutant concentrations and month of the year. Pollutant concentrations were divided by the median to obtain comparable scales.

Air pollutant concentrations exhibited considerable variation over time. Pronounced seasonal changes were observed, such as increased heating use in the winter, contributing to higher pollutant levels. Additionally, significant weekly variations were noted, likely due to different mobility patterns on weekends. These weekly fluctuational changes are depicted in Figure 1A, indicating increased levels of all measured air pollutants, except ozone, during weekdays, with a reduction during the weekend that extends into Monday. Seasonal trends are displayed in Figure 1B, indicating that, whilst most ambient pollutants peak during winter, ozone levels are highest in summer. Specifically, these average seasonal variations amounted to 22.1 ppm (132.4%) for PM_2.5_, 23.76 ppm (106.4%) for PM_10_, 0.66 ppm (189.7%) for CO, 25.6 ppm (99.6%) for NO_2_, 48.5 ppm (217.9%) for NOx, 2.6 ppm (113.5%) for SO_2_ and 62 ppm (137.8%) for ozone. Notably, the incidence of myocardial infarction also varied between the weekends (2.9 per day), Mondays (4.7 per day) and other workdays (4.1 per day). These variations are incorporated in the statistical model used to analyze all further effects.

**Figure 2:**
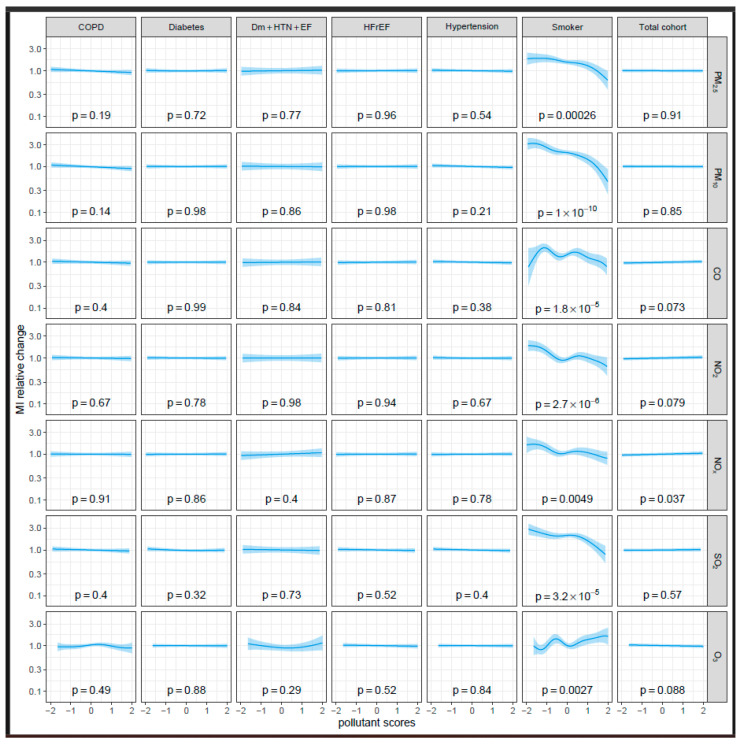
The interaction effect of pollutants modeled by a spline curve in a Poisson regression model. The interaction effect is the change in the effect of the pollutant that can be attributed to the risk factor. All pollutants except ozone were transformed logarithmically and all pollutants were standardized to a mean of zero and a standard deviation of one.

To evaluate the effects of comorbidities and risk factors on the augmentation of risk, we analyzed the impact of various cardiovascular risk factors: diabetes mellitus (*n* = 3111, 24.7%), arterial hypertension (*n* = 9235, 73.4%), heart failure with reduced ejection fraction (HFrEF) (*n* = 3010, 38.1%) and current smoking (*n* = 665, 8.0%), as well as the presence of COPD (*n* = 1170, 9.3%). As shown in Figure 2, none of the analyzed pollutants affected the relative risk of MI in the total cohort (left column) or in the subgroups with these risk factors. Specifically, the lowest *p*-values calculated for the relative change in MI frequency were 0.32 for diabetes and SO_2_, 0.21 for hypertension and PM_10_ and 0.36 for HFrEF and SO_2_—thus, are far from statistical significance. Even among patients with a combination of diabetes mellitus, arterial hypertension and HFrEF, who have previously been described as the most vulnerable, did not exhibit a significantly increased risk with elevated ambient air pollution compared to patients with none of the three (*n* = 848, 41.8%) (see Table 2). In patients with chronic lung diseases, such as COPD, that are considered highly vulnerable to air pollution, only a non-significant trend toward decreased risk was noted for PM_2.5_ (*p* = 0.19) and PM_10_ (*p* = 0.14), with virtually no change observed for any of the other pollutants. 

Current smoking was the only factor that significantly impacted the risk of myocardial infarction with increasing levels of ambient air pollution. Highly significant decreases in the relative risk for myocardial infarction are shown for PM_2.5_ and PM_10_, for NO_2_, NOx and SO_2_ and for CO at higher concentrations (*p* < 0.01 for all pollutants). In contrast, airborne ozone levels were positively associated with an increased risk among current smokers, with a significant rise in risk at higher ozone concentrations (*p* = 0.0027).

These findings were confirmed in the conditional logistic case-crossover model (see Supplemental Figure S1 and Supplemental Table S1).

**Figure 3:**
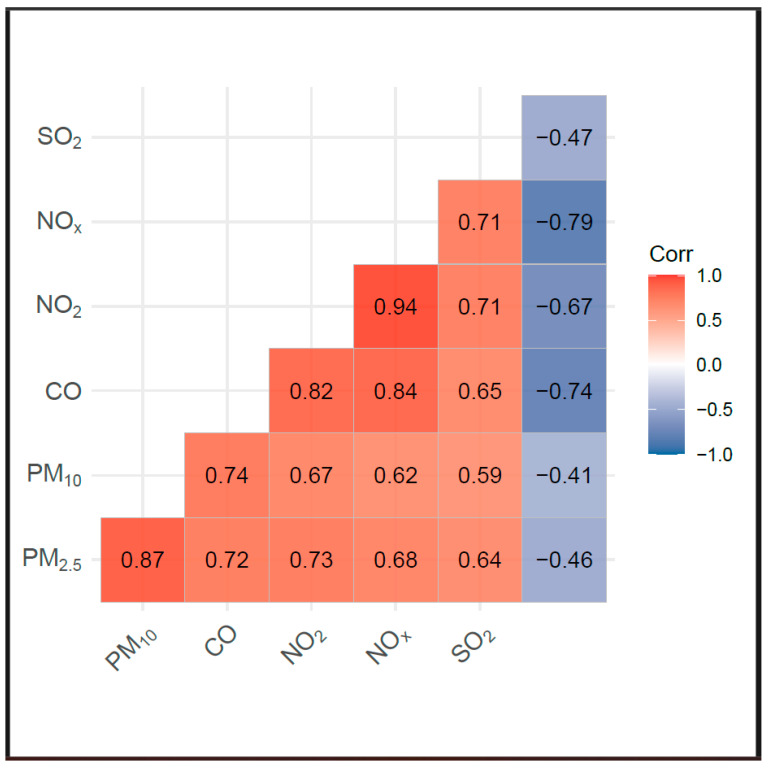
Spearman’s correlation of pollutants after adjusting for day of week and month of year.

Interpreting the effects of air pollution requires considering the interactions among different air pollutants. As plotted in the Spearman’s correlation matrix in Figure 3, a positive correlation exists between PM_2.5_, PM_10_, CO, NO_2_, NOx and SO_2_, whereas O_3_ has a negative correlation with these pollutants.

**Figure 4:**
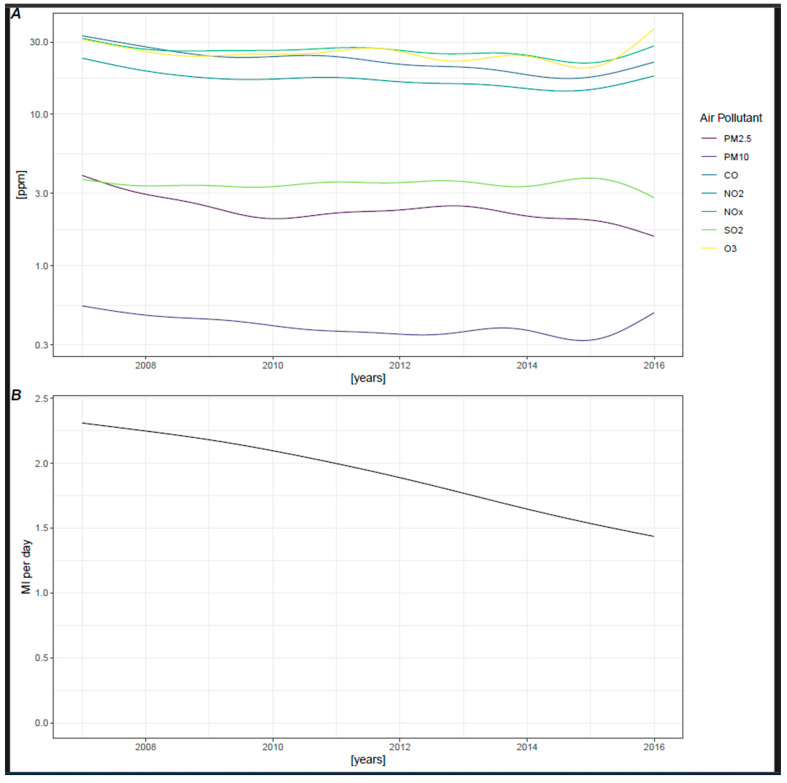
(**A**) Trends in pollutant concentration with spline interpolation. (**B**) Trend of MI per day with spline interpolation in Poisson model.

Lastly, air pollution is subject to long-term changes: as illustrated in Figure 4A, concentrations of all air pollutants tended to decrease over the years. Correspondingly, the incidence of myocardial infarctions also significantly declined during the observation period, as shown in Figure 4B.

## 4. Discussion

Without doubt, air pollution is a relevant public health issue that also adversely affects cardiovascular health. According to a recent Global Burden of Disease (GBD) study, ambient air pollution caused 4.2 million deaths (7.6% of total global mortality) and 103.1 million disability-adjusted life years (DALYs) (4.2% of global DALYs) in 2015 [22].

Available reviews indicate a positive association between short-term exposure to PM and NOx and the incidence of MI [23]. However, the most significant results have been reported in low- and medium-income countries, where air pollution is typically higher compared to most high-income countries [23]. However, it needs to be acknowledged that even positive studies showing a significant impact of air pollutants on the incidence of myocardial infarction only report absolute overall increases between 0.5% and a maximum of 5% [11]. Several studies have tested various pollutants, multiple lag times and different comorbidities. Generally, these studies identify significant associations only within specific subsets, during particular lag times [8,10] or only for certain tested comorbidities. Furthermore, it is often unclear whether these analyses are limited to the results shown in the manuscripts, if more tests were conducted or whether a robust correction for multiple testing was applied.

Additionally, the association between air pollutants and an increased risk of myocardial infarction seems to be limited to lower ranges of air pollution. Pollution levels exceeding certain thresholds—specifically, a PM_2.5_ cut-off of 33.63 µg/m^3^ and PM_10_ cut-off of 57.3 µg/m^3^ [8]—did not result in a further increased risk for MI. This finding is particularly significant given the considerable variability in air pollution levels between studies.

The widely accepted main mechanism of ambient air pollution harming the cardiovascular system involves enhanced endothelial and smooth muscle inflammation. The first evidence of this was that SGLT2 inhibitors might attenuate the inflammatory response in human cell models [24]. This might be of particular interest, as augmenting data was recently published using SGLT2 inhibitors in the setting of myocardial infarction. The EMMY trial was the first clinical trial highlighting a significant reduction in NTproBNP levels, as well as in functional and structural cardiac parameters, for Empagliflozin compared to a placebo [25] and confirmed this finding to be independent of sex [26], diabetic status [27], baseline ECG [28] or the timing of SGLT2i initiation [29]. 

Moreover, the recently published DAPA-MI trial was the first clinical outcome trial powered for hard clinical endpoints to report significant beneficial effects on cardiometabolic outcomes for Dapagliflozin compared to a placebo; however, no impact on the composite of cardiovascular death or hospitalization for heart failure compared with a placebo was observed post-MI [30]. For Empagliflozin, another large outcome trial (EMPACT-MI) was published indicating neutral effects on the primary endpoint of all-cause death and hospitalization for heart failure, although the secondary endpoint of heart failure hospitalizations revealed a numerically lower number in the SGLT2-I group [31]. However, due to the lack of significant reductions in hard clinical endpoints, a general indication for using SGLT2i in ACS patients cannot be recommended [32].

Besides these aspects, it is crucial to consider the potential for publication bias. Therefore, data should be interpreted carefully to avoid overinterpretations.

Nonetheless, our analysis revealed a highly significant association between air pollution and smoking status. Counterintuitively, higher levels of ambient air pollution levels were associated with a lower relative risk for myocardial infarction in current smokers, except for ozone, which showed a positive association in this subgroup. Although this finding may seem counterintuitive, it aligns with previously published data reporting a significantly elevated risk associated with increased concentrations of air particulate matter for non-smokers only [12,19] in an analysis of 1354 patients experiencing their first incidence of STEMI. It can be speculated that active smokers, already chronically exposed to severely reduced air quality and high pollutants, may not experience a significant increase in risk from additional ambient air pollution. Conversely, non-smokers, having a lower baseline of exposure, respond more sensitively. Additionally, multifactorial effects, including socio-economic, psychological or nutritional factors, might play a role. As smoking is considered to be a key factor in the so called “exposome” [33] but correlates with multiple other health-impacting factors, it might also be a coincidence in exposing factors. However, not smoking alone accounted for the different findings in this subpopulation. 

### Study Limitations

First, air pollution measurements were conducted at testing stations within Graz, the capital of Styria. Although the majority of inhabitants in Styria live in and around Graz, a portion reside in more rural and mountainous areas where air pollution concentrations might differ. As correlations of pollution concentrations between the different areas in Styria cannot be reliably drawn, no adjustments were made for the presented data. 

Second, exposures to some of the studied air pollutants were highly correlated. This correlation limits our ability to conduct multipollutant models and complicates the distinction of their respective effects on MI incidence. 

Third, as with any observational study, despite the case-crossover design accounting for time-variant confounding factors, the potential for residual or unmeasured confounding factors still persists. 

Fourth, given that our findings were obtained from a single province in Austria, caution should be exercised in generalizing these findings to populations in other regions or countries.

Fifth, the potential effect of smoking to differ the effect of ambient air pollution on cardiovascular health does not trivialize the undoubted negative general health impact of smoking.

## Figures and Tables

**Table 1:** Subgroup characteristics. Age and body mass index (BMI) shown as inter-quartile ranges (IQR). COPD: chronic obstructive pulmonary disease, HFrEF: heart failure with reduced ejection fraction, DM: diabetes mellitus, HTN: arterial hypertension.

Risk Factors	*n*	Sex (Male)	Sex (Female)	Age (IQR)	BMI (IQR)	STEMI	NSTE-ACS
All	12,581	8192 (65.1%)	4389 (34.9%)	68 (58–76)	27 (24.4–30.1)	4217 (33.5%)	8364 (66.5%)
COPD	1170	796 (68%)	374 (32%)	71 (64–78)	27.3 (24.2–30.2)	254 (21.7%)	916 (78.3%)
Diabetes	3111	1921 (61.7%)	1190 (38.3%)	71 (63–77)	28.4 (25.5–31.7)	862 (27.7%)	2249 (72.3%)
HFrEF	3010	1862 (61.9%)	1148 (38.1%)	73 (64–80)	26.2 (23.9–29.1)	1080 (35.9%)	1930 (64.1%)
Hypertension	9235	5837 (63.2%)	3398 (36.8%)	69 (60–77)	27.4 (24.8–30.5)	2724 (29.5%)	6511 (70.5%)
Smoker	665	516 (77.6%)	149 (22.4%)	56 (49–64)	27.2 (24.4–30.4)	324 (48.7%)	341 (51.3%)
NSTE-ACS	8364	5244 (62.7%)	3120 (37.3%)	69 (60–77)	27.1 (24.4–30.1)	0 (0%)	8364 (100%)
STEMI	4217	2948 (69.9%)	1269 (30.1%)	65 (54–75)	26.8 (24.4–29.7)	4217 (100%)	0 (0%)
No Dm, HTN, HFrEF	1181	877 (74.3%)	304 (25.7%)	58 (50–68)	25.9 (23.8–28.7)	551 (46.7%)	630 (53.3%)
DM + HTN + HFrEF	848	513 (60.5%)	335 (39.5%)	74 (67–80)	27.7 (25–31.1)	240 (28.3%)	608 (71.7%)

**Table 2:** Relative interaction effects of the comorbidities and smoking with pollutants on the incidence of MI in the Poisson regression. The effect relates to the relative increase in MI risk if the pollutant is increased by one standard deviation relative to a person without the comorbidity or a non-smoker, and 95% confidence limits are given in brackets. All pollutants except ozone were transformed by the base ten logarithm function. In these cases, the effect relates to the tenfold concentration of the pollutant. CO: carbon monoxide, COPD: chronic obstructive pulmonary disease, Dm + HTN + EF: diabetes mellitus + hypertension + heart failure with reduced ejection fraction, HFrEF: heart failure with reduced ejection fraction, STEMI: ST-elevation myocardial infarction, NO_2_: nitrogen dioxide, NOx: nitrogen oxides, O_3_: ozone, PM_10_: particulate matter up to 10 μm in size, PM_2.5_: particulate matter up to 2.5 μm in size, SO_2_: sulfur dioxide. Std.Err: standard error of the mean; Confident.Rel: confidential interval.

Disease	Diseased	Not Diseased	Pollutant	Coefficient	Std.Err	Confint.Rel	*p*-Value
COPD	1170	11,411	CO	−0.032	0.066	0.969 (0.851–1.103)	0.63
			NO_2_	0.005	0.079	1.005 (0.861–1.173)	0.95
			NOx	0.042	0.068	1.042 (0.912–1.192)	0.54
			O_3_	−0.184	0.081	0.832 (0.71–0.975)	0.023
			PM_10_	0.025	0.071	1.025 (0.892–1.177)	0.73
			PM_2.5_	0.076	0.072	1.078 (0.936–1.243)	0.3
			SO_2_	−0.046	0.07	0.955 (0.834–1.095)	0.51
Diabetes	3111	9470	CO	0.047	0.045	1.048 (0.96–1.144)	0.3
			NO_2_	−0.015	0.052	0.986 (0.889–1.092)	0.78
			NOx	0.044	0.046	1.045 (0.955–1.145)	0.34
			O_3_	0.035	0.054	1.036 (0.932–1.151)	0.51
			PM_10_	0.027	0.047	1.027 (0.937–1.126)	0.57
			PM_2.5_	0.061	0.048	1.063 (0.967–1.168)	0.21
			SO_2_	0.036	0.047	1.037 (0.946–1.137)	0.44
Dm + HTN + HFrEF	848	1181	CO	0.134	0.097	1.143 (0.946–1.382)	0.17
			NO_2_	−0.09	0.113	0.914 (0.733–1.141)	0.43
			NOx	0.112	0.099	1.118 (0.922–1.357)	0.26
			O_3_	0.237	0.118	1.268 (1.007–1.596)	0.044
			PM_10_	0.006	0.101	1.006 (0.826–1.226)	0.95
			PM_2.5_	0.084	0.104	1.087 (0.887–1.333)	0.42
			SO_2_	−0.002	0.104	0.998 (0.813–1.224)	0.98
HFrEF	3010	4894	CO	0.033	0.051	1.034 (0.936–1.142)	0.51
			NO_2_	−0.07	0.059	0.932 (0.83–1.048)	0.24
			NOx	0.012	0.052	1.012 (0.915–1.121)	0.81
			O_3_	−0.049	0.06	0.952 (0.846–1.072)	0.42
			PM_10_	−0.009	0.054	0.991 (0.892–1.102)	0.87
			PM_2.5_	0.02	0.055	1.02 (0.915–1.137)	0.72
			SO_2_	0.078	0.054	1.081 (0.972–1.202)	0.15
Hypertension	9235	3346	CO	0.025	0.044	1.025 (0.941–1.117)	0.57
			NO_2_	0.018	0.052	1.018 (0.92–1.127)	0.73
			NOx	0.013	0.045	1.013 (0.928–1.107)	0.77
			O_3_	0.028	0.053	1.028 (0.927–1.141)	0.59
			PM_10_	−0.038	0.046	0.963 (0.879–1.054)	0.41
			PM_2.5_	0.017	0.048	1.017 (0.926–1.117)	0.72
			SO_2_	−0.062	0.046	0.94 (0.86–1.028)	0.18
Smoker	665	7677	CO	−0.445	0.111	0.641 (0.515–0.797)	0.000066
			NO_2_	−0.145	0.119	0.865 (0.685–1.092)	0.22
			NOx	−0.253	0.102	0.777 (0.636–0.948)	0.013
			O_3_	0.272	0.105	1.313 (1.068–1.612)	0.0096
			PM_10_	−0.674	0.139	0.51 (0.388–0.669)	0.0000012
			PM_2.5_	−0.408	0.123	0.665 (0.522–0.847)	0.00096
			SO_2_	−0.635	0.13	0.53 (0.411–0.683)	0.000001
STEMI	4217	8364	CO	−0.053	0.042	0.948 (0.874–1.029)	0.2
			NO_2_	−0.129	0.049	0.879 (0.799–0.967)	0.0082
			NOx	−0.096	0.042	0.909 (0.836–0.988)	0.024
			O_3_	−0.038	0.049	0.963 (0.874–1.06)	0.44
			PM_10_	−0.032	0.044	0.968 (0.888–1.055)	0.46
			PM_2.5_	−0.052	0.045	0.949 (0.869–1.037)	0.25
			SO_2_	−0.057	0.044	0.945 (0.868–1.029)	0.19

## Data Availability

Due to ethics board rules data can only be made available by the corresponding author upon reasonable request.

## Supplementary Materials

The following supporting information can be downloaded at: https://www.mdpi.com/article/10.3390/jcm13237324/s1, Figure S1: Conditional logistic case cross-over model; Table S1: Relative interaction effects of comorbidities and smoking with pollutants on the incidence of MI in conditional logistic case cross-over regression. The effect relates to the relative increase of the odds of MI if the pollutant is increased by one standard deviaton relative to days of the week of the same month without the comorbidity or a non-smoker. All pollutants except ozone were transformed by the base ten logarithm function. In these cases the effect relates to the tenfold concentration of the pollutant. 95% confidence limits are given in brackets. CO: Carbon Monoxide, COPD: Chronic Obstructive Pulmonary Disease, Dm + HTN + EF: Diabetes mellitus + Hypertension + Heart Failure with reduced Ejection Fraction, HFrEF: Heart Failure with reduced Ejection Fraction, NO_2_: Nitrogen Dioxide, NO_x_: Nitrogen Oxides, O_3_: Ozone, PM_10_: Particulate Matter up to 10 micrometers in size, PM_2.5_: Particulate Matter up to 2.5 micrometers in size, SO_2_: Sulfur Dioxide.
