# Supplementary material for: Air Pollution and Myocardial Infarction—A New Smoker’s Paradox?
Source: J Clin Med. 2024 Dec 2;13(23):7324. doi: 10.3390/jcm13237324 (PMC11642313; doi:10.3390/jcm13237324)
Supplement: Supplementary file 1 [file jcm-13-07324-s001.zip › jcm-3306611-supplementary.docx]

**Supplementary Materials**

**Table S1.** Relative interaction effects of comorbidities and smoking with pollutants on the incidence of MI in conditional logistic case cross-over regression. The effect relates to the relative increase of the odds of MI if the pollutant is increased by one standard deviaton relative to days of the week of the same month without the comorbidity or a non-smoker. All pollutants except ozone were transformed by the base ten logarithm function. In these cases the effect relates to the tenfold concentration of the pollutant. 95% confidence limits are given in brackets. CO: Carbon Monoxide, COPD: Chronic Obstructive Pulmonary Disease, Dm+HTN+EF: Diabetes mellitus + Hypertension + Heart Failure with reduced Ejection Fraction, HFrEF: Heart Failure with reduced Ejection Fraction, NO2: Nitrogen Dioxide, NOx: Nitrogen Oxides, O3: Ozone, PM10: Particulate Matter up to 10 micrometers in size, PM2.5: Particulate Matter up to 2.5 micrometers in size, SO2: Sulfur Dioxide.

| **Disease** | **diseased** | **not diseased** | **pollutant** | **coefficient** | **std.err** | **confint.rel** | **p-value** |
| --- | --- | --- | --- | --- | --- | --- | --- |
| COPD | 1170 | 11411 | CO | -0.025 | 0.03 | 0.975 (0.919 - 1.035) | 0.41 |
|  |  |  | NO2 | -0.013 | 0.03 | 0.987 (0.93 - 1.048) | 0.67 |
|  |  |  | NOx | -0.004 | 0.03 | 0.996 (0.939 - 1.057) | 0.91 |
|  |  |  | O3 | 0.006 | 0.031 | 1.006 (0.947 - 1.069) | 0.85 |
|  |  |  | PM10 | -0.045 | 0.03 | 0.956 (0.901 - 1.015) | 0.14 |
|  |  |  | PM2.5 | -0.039 | 0.03 | 0.962 (0.906 - 1.021) | 0.2 |
|  |  |  | SO2 | -0.026 | 0.031 | 0.974 (0.918 - 1.035) | 0.4 |
| Diabetes | 3111 | 9470 | CO | 0.001 | 0.02 | 1.001 (0.962 - 1.041) | 0.97 |
|  |  |  | NO2 | -0.006 | 0.02 | 0.994 (0.955 - 1.035) | 0.78 |
|  |  |  | NOx | 0.004 | 0.02 | 1.004 (0.964 - 1.045) | 0.86 |
|  |  |  | O3 | -0.003 | 0.021 | 0.997 (0.957 - 1.038) | 0.87 |
|  |  |  | PM10 | 0 | 0.02 | 1 (0.961 - 1.041) | 0.99 |
|  |  |  | PM2.5 | 0 | 0.02 | 1 (0.961 - 1.041) | 0.99 |
|  |  |  | SO2 | -0.017 | 0.021 | 0.984 (0.945 - 1.024) | 0.42 |
| Dm+HTN+EF | 848 | 1181 | CO | 0.009 | 0.045 | 1.009 (0.925 - 1.102) | 0.83 |
|  |  |  | NO2 | 0.002 | 0.045 | 1.002 (0.918 - 1.093) | 0.97 |
|  |  |  | NOx | 0.038 | 0.045 | 1.038 (0.951 - 1.133) | 0.4 |
|  |  |  | O3 | -0.021 | 0.046 | 0.979 (0.896 - 1.071) | 0.65 |
|  |  |  | PM10 | -0.008 | 0.045 | 0.992 (0.908 - 1.084) | 0.86 |
|  |  |  | PM2.5 | 0.013 | 0.045 | 1.013 (0.928 - 1.107) | 0.77 |
|  |  |  | SO2 | -0.016 | 0.045 | 0.984 (0.9 - 1.076) | 0.72 |
| HFrEF | 3010 | 4894 | CO | 0.007 | 0.023 | 1.007 (0.963 - 1.054) | 0.75 |
|  |  |  | NO2 | 0.003 | 0.023 | 1.003 (0.959 - 1.049) | 0.89 |
|  |  |  | NOx | 0.004 | 0.023 | 1.004 (0.959 - 1.05) | 0.87 |
|  |  |  | O3 | -0.015 | 0.023 | 0.985 (0.941 - 1.031) | 0.52 |
|  |  |  | PM10 | 0.001 | 0.023 | 1.001 (0.957 - 1.047) | 0.97 |
|  |  |  | PM2.5 | 0.002 | 0.023 | 1.002 (0.957 - 1.048) | 0.95 |
|  |  |  | SO2 | -0.015 | 0.023 | 0.985 (0.94 - 1.031) | 0.51 |
| Hypertension | 9235 | 3346 | CO | -0.018 | 0.02 | 0.983 (0.945 - 1.022) | 0.38 |
|  |  |  | NO2 | -0.007 | 0.02 | 0.993 (0.955 - 1.033) | 0.74 |
|  |  |  | NOx | 0.006 | 0.02 | 1.006 (0.967 - 1.046) | 0.78 |
|  |  |  | O3 | -0.004 | 0.02 | 0.996 (0.957 - 1.036) | 0.83 |
|  |  |  | PM10 | -0.025 | 0.02 | 0.975 (0.938 - 1.014) | 0.21 |
|  |  |  | PM2.5 | -0.012 | 0.02 | 0.988 (0.95 - 1.027) | 0.53 |
|  |  |  | SO2 | -0.021 | 0.02 | 0.979 (0.941 - 1.019) | 0.3 |
| Smoker | 665 | 7677 | CO | -0.208 | 0.042 | 0.812 (0.747 - 0.882) | 0.00000092 |
|  |  |  | NO2 | -0.202 | 0.039 | 0.817 (0.757 - 0.883) | 0.00000025 |
|  |  |  | NOx | -0.145 | 0.041 | 0.865 (0.798 - 0.937) | 0.0004 |
|  |  |  | O3 | 0.143 | 0.04 | 1.154 (1.066 - 1.249) | 0.00041 |
|  |  |  | PM10 | -0.302 | 0.04 | 0.739 (0.683 - 0.8) | 0.000000000000067 |
|  |  |  | PM2.5 | -0.188 | 0.04 | 0.829 (0.766 - 0.897) | 0.0000028 |
|  |  |  | SO2 | -0.21 | 0.04 | 0.811 (0.75 - 0.876) | 0.00000013 |
| STEMI | 4217 | 8364 | CO | -0.013 | 0.019 | 0.987 (0.952 - 1.024) | 0.48 |
|  |  |  | NO2 | -0.03 | 0.019 | 0.97 (0.935 - 1.007) | 0.11 |
|  |  |  | NOx | -0.02 | 0.019 | 0.98 (0.945 - 1.016) | 0.27 |
|  |  |  | O3 | 0.013 | 0.019 | 1.013 (0.976 - 1.052) | 0.49 |
|  |  |  | PM10 | -0.036 | 0.019 | 0.965 (0.93 - 1.001) | 0.056 |
|  |  |  | PM2.5 | -0.029 | 0.019 | 0.971 (0.936 - 1.007) | 0.12 |
|  |  |  | **SO2** | **-0.057** | **0.044** | **0.945 (0.868 - 1.029)** | **0.19** |


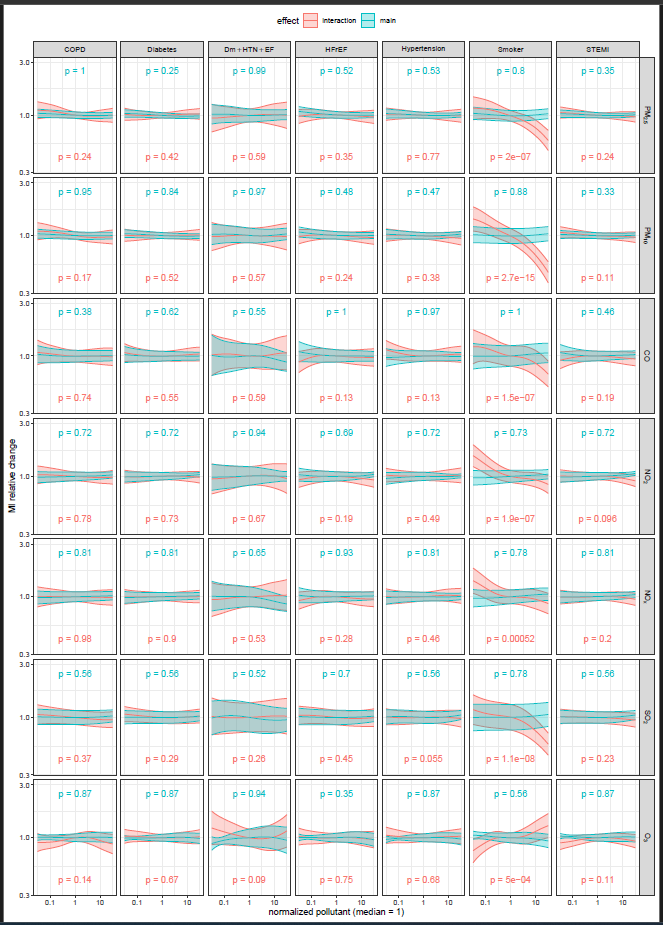


**Figure S1.** Conditional logistic case cross-over model.
